# Supplementary material for: The miR-144/Hmgn2 regulatory axis orchestrates chromatin organization during erythropoiesis
Source: Nat Commun. 2024 May 7;15:3821. doi: 10.1038/s41467-024-47982-2 (PMC11076586; doi:10.1038/s41467-024-47982-2)
Supplement: Supplementary file 1 — Supplementary Information [file 41467_2024_47982_MOESM1_ESM.pdf]

SUPPLEMENTARY INFORMATION

The miR-144/Hmgn2 regulatory axis orchestrates chromatin organization during erythropoiesis.

Dmitry A. Kretov[1], Leighton Folkes[2], Alexandra Mora-Martin[1], Isha A. Walawalkar[1], Imrat[1], Noreen Syedah[1], Kim Vanuytsel[3,4,5], Simon Moxon[2], George J. Murphy[3,4], Daniel Cifuentes[1,6,†,*]

[1]Department of Biochemistry and Cell Biology, Boston University Chobanian & Avedisian School of Medicine, Boston, MA

[2]School of Biological Sciences, University of East Anglia, Norwich, UK

[3]Center for Regenerative Medicine, Boston University Chobanian & Avedisian School of Medicine, Boston, MA

[4]Section of Hematology and Oncology, Department of Medicine, Boston Medical Center, Boston, Massachusetts, USA

[5]Amyloidosis Center, Boston University Chobanian and Avedisian School of Medicine, Boston, Massachusetts, USA

[6]Department of Virology, Immunology and Microbiology, Boston University Chobanian & Avedisian School of Medicine, Boston, MA

[†]Lead contact: Daniel Cifuentes

*Correspondence: dcb@bu.edu

Figure S1

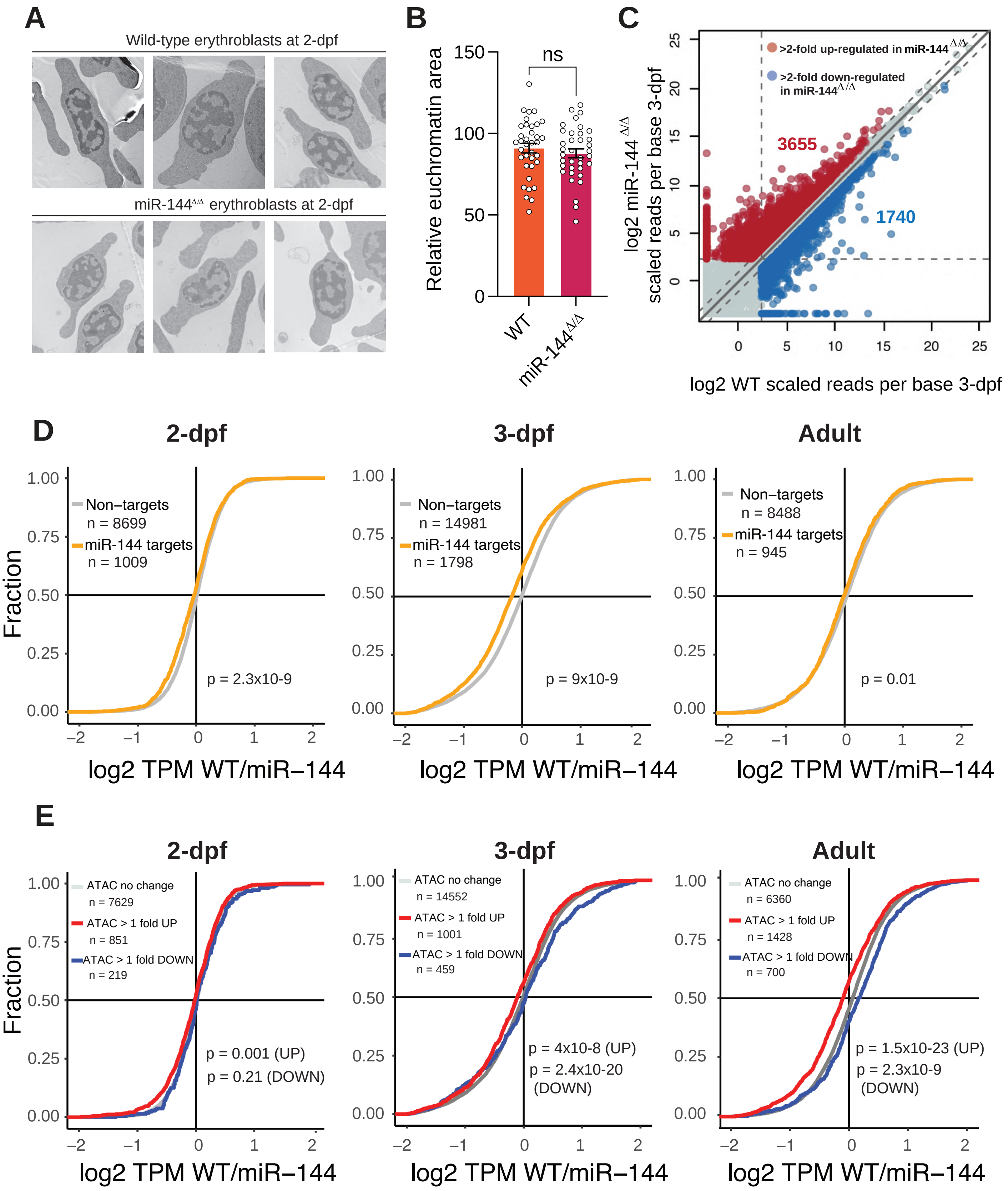

A
Wild-type erythroblasts at 2-dpf
miR-144Δ/Δ erythroblasts at 2-dpf
B
Relative euchromatin area
ns
WT
miR-144Δ/Δ
C
>2-fold up-regulated in miR-144Δ/Δ
>2-fold down-regulated in miR-144Δ/Δ
3655
1740
log2 miR-144Δ/Δ scaled reads per base 3-dpf
log2 WT scaled reads per base 3-dpf
D
2-dpf
3-dpf
Adult
Fraction
Non–targets n = 8699
miR-144 targets n = 1009
p = 2.3x10-9
Non–targets n = 14981
miR-144 targets n = 1798
p = 9x10-9
Non–targets n = 8488
miR-144 targets n = 945
p = 0.01
log2 TPM WT/miR–144
E
2-dpf
3-dpf
Adult
Fraction
ATAC no change n = 7629
ATAC > 1 fold UP n = 851
ATAC > 1 fold DOWN n = 219
p = 0.001 (UP)
p = 0.21 (DOWN)
ATAC no change n = 14552
ATAC > 1 fold UP n = 1001
ATAC > 1 fold DOWN n = 459
p = 4x10-8 (UP)
p = 2.4x10-20 (DOWN)
ATAC no change n = 6360
ATAC > 1 fold UP n = 1428
ATAC > 1 fold DOWN n = 700
p = 1.5x10-23 (UP)
p = 2.3x10-9 (DOWN)
log2 TPM WT/miR–144

Figure S1. Related to Main Figure 1. Cellular, transcriptomic, and genomic effects of miR-144 loss during erythropoiesis.

(A) Transmission Electron Microscopy of erythrocytes isolated from 2-dpf embryos.

(B) Quantification of euchromatic regions of the nuclei from (A). Individual cells (n=35) are analyzed in each case. *p*-values from two-tailed unpaired *t*-test. Error bars represent standard error of the mean.

(C) RNA-Sequencing of erythrocytes isolated from 3-dpf from miR-144$^{\Delta/\Delta}$ zebrafish and wild-type siblings. Average of 2 biological replicates is plotted. Each biological replicate represents the peripheral blood extracted from pools of ~100 embryos from different clutches.

(D) Cumulative distributions of fold changes revealed by RNA-Sequencing between wild-type and miR-144$^{\Delta/\Delta}$ and wild-type erythrocytes for curated miR-144 targets (expressed more than 5 scaled reads per base in erythrocytes and have predicted miR-144 targets sites according to TargetScan (red line) and non-targets (grey).

(E) Cumulative distributions of fold changes of gene expression revealed by RNA-Sequencing between miR-144$^{\Delta/\Delta}$ and wild-type erythrocytes for the genes with increased more than 1-fold (red line), decreased more than 1-fold (blue line) and not changed (gray line) chromatin accessibility in genes.

Figure S2

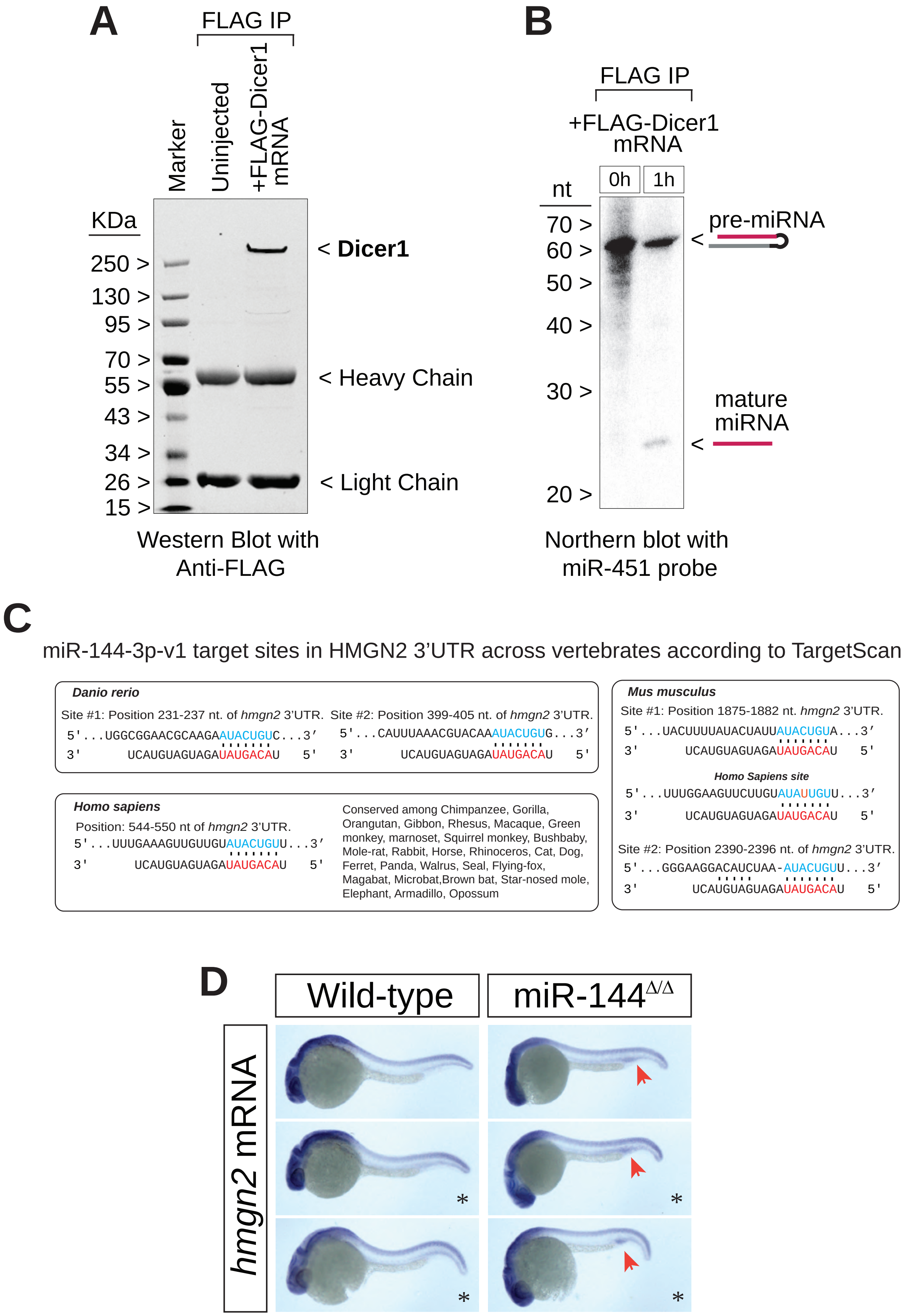
A
FLAG IP
Marker
Uninjected
+FLAG-Dicer1 mRNA
KDa
250 >
130 >
95 >
70 >
55 >
43 >
34 >
26 >
15 >
< Dicer1
< Heavy Chain
< Light Chain
Western Blot with Anti-FLAG
B
FLAG IP
+FLAG-Dicer1 mRNA
nt
0h
1h
70 >
60 >
50 >
40 >
30 >
20 >
pre-miRNA
mature miRNA
Northern blot with miR-451 probe
C
miR-144-3p-v1 target sites in HMGN2 3'UTR across vertebrates according to TargetScan
Danio rerio
Site #1: Position 231-237 nt. of hmgn2 3'UTR.
5'...UGGCGGAACGCAAGAAUACUGUC...3'
3' UCAUGUAGUAGAUAUGACAU 5'
Site #2: Position 399-405 nt. of hmgn2 3'UTR.
5'...CAUUUAAACGUACAAAUACUGUG...3'
3' UCAUGUAGUAGAUAUGACAU 5'
Homo sapiens
Position: 544-550 nt of hmgn2 3'UTR.
5'...UUUGAAAGUUGUUGUAUACUGUU...3'
3' UCAUGUAGUAGAUAUGACAU 5'
Conserved among Chimpanzee, Gorilla, Orangutan, Gibbon, Rhesus, Macaque, Green monkey, marnoset, Squirrel monkey, Bushbaby, Mole-rat, Rabbit, Horse, Rhinoceros, Cat, Dog, Ferret, Panda, Walrus, Seal, Flying-fox, Magabat, Microbat,Brown bat, Star-nosed mole, Elephant, Armadillo, Opossum
Mus musculus
Site #1: Position 1875-1882 nt. hmgn2 3'UTR.
5'...UACUUUUAUACUAUUAUACUGUA...3'
3' UCAUGUAGUAGAUAUGACAU 5'
Homo Sapiens site
5'...UUUGGAAGUUCUUGUAUAUUGUU...3'
3' UCAUGUAGUAGAUAUGACAU 5'
Site #2: Position 2390-2396 nt. of hmgn2 3'UTR.
5'...GGGAAGGACAUCUAA-AUACUGUU...3'
3' UCAUGUAGUAGAUAUGACAU 5'
D
Wild-type
miR-144Δ/Δ
hmgn2 mRNA
*

Figure S2. Related to Main Figure 2.

(A) Western blot showing the expression of Dicer1 in injected embryos. ~100 embryos injected with an *in vitro* transcribed mRNA encoding FLAG-Dicer1 are subjected to pull-down with FLAG antibody linked to magnetic beads and then resolved in a Western blot with FLAG antibody.

(B) Dicing assay. FLAG-Dicer1 expressed in embryos injected with mRNA encoding FLAG-Dicer1 is pull-down with FLAG-antibody coated beads. Then the bead pellet is used to process a microRNA precursor, in this case pre-miR-451 that has been engineered to be Dicer-dependent. The Northern blot with a miR-451 probe shows that at the initial time point only the microRNA precursor is present, while one hour later we can also observe the mature miRNA, the product of Dicer-mediated cleavage.

(C) Predicted miR-144-3p-v1 (according to TargetScan target sites in *Hmgn2* 3'UTR of *Danio rerio*, *Homo sapiens* and *Mus musculus*. miR-144 seed region is indicated in red.

(D) (Related to Fig. 2D) Whole-mount *in situ* hybridization of *hmgn2* mRNA at 30-hpf of miR-144$^{\Delta/\Delta}$ fish and wild-type siblings. The embryos presented in the figure are representative of an experiment with n=17 embryos of each genotype. Blue staining indicates the presence of *hmgn2* mRNA. Red arrowhead points to the area where *hmgn2* mRNA accumulates upon the loss of miR-144. A close-up cropped image is shown in main Figure 2D for the embryos marked with an asterisk.

Figure S3

A UMAP plot colored by genotype

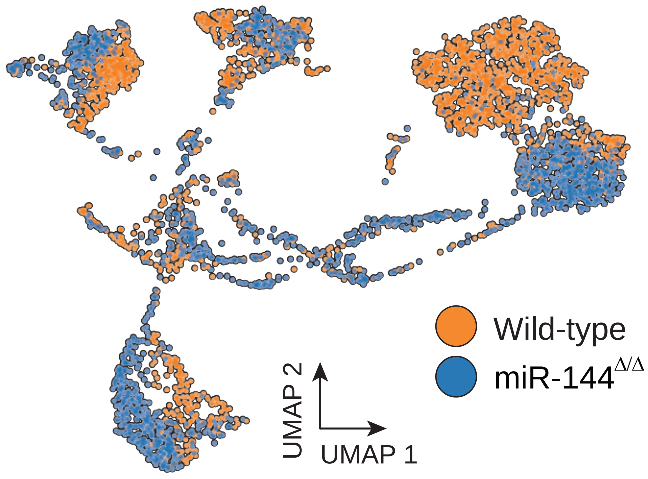

B Cluster connectivity assigned with PAGA

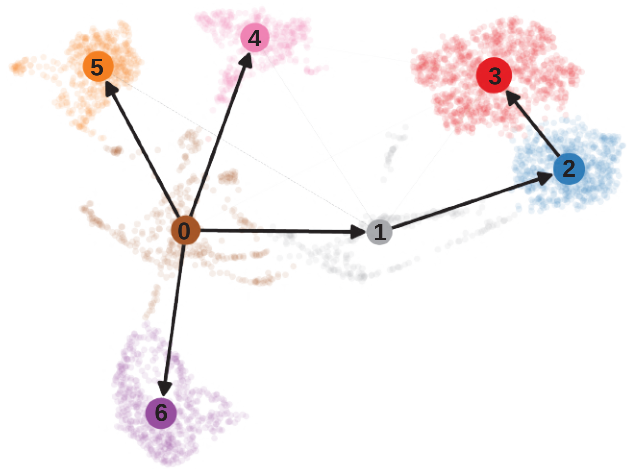

C Cluster assignment to hematopoietic lineages

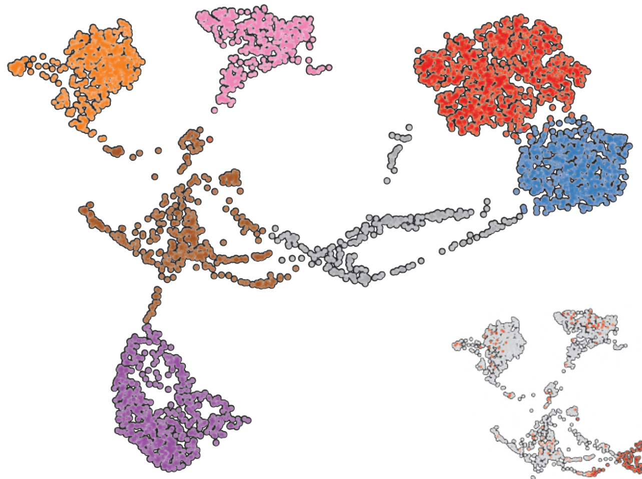

		Cells per cluster	
		WT	miR-144$^{\Delta/\Delta}$
Hematopoietic progenitors	0	170	248
Early erythrocytes	1	57	192
Intermediate erythrocytes	2	133	468
Mature erythrocytes	3	943	45
T-cells	4	243	168
B-cells	5	309	205
Neutrophils	6	179	375
Total =		2,034	1,701

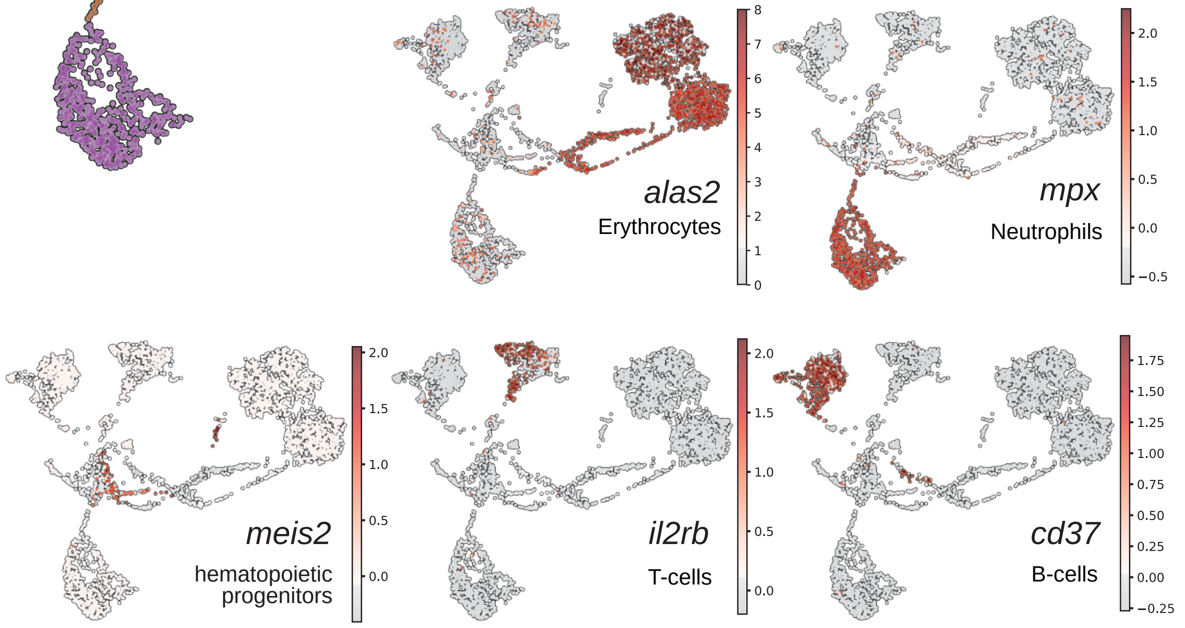

Figure S3. Related to Main Figure 2. Detailed analysis of scRNA-Seq data of hematopoietic tissue from wild-type and miR-144 mutant embryos.

(A) UMAP of scRNA-Seq data from zebrafish pronephros. Each dot represents a single cell. The plot is colored according to the genotype of origin. The data is collected from 1 wild-type pronephros and 1 miR-144 mutant pronephros.

(B) UMAP plot showing developmental trajectories between clusters. Developmental trajectories were inferred using scVelo and plotted (black arrows) applying partition-based graph abstraction (PAGA).

(C) Cluster assignment to the major hematopoietic lineages. UMAP is colored according to the expression levels of marker genes representative of each main hematopoietic cell lineage. Table shows number of cells in each cluster according to the genotype of origin.

Figure S4

A

```
               1
HMGN2-WT       MPKRKIDGEKGAKGKEEPTRRSARLSAKPAPPKPEPKAK
HMGN2 △12      MPKRK------------PTRRSARLSAKPAPPKPEPKAK

               40
HMGN2-WT       KAAPKKAAKGKKGSNPAENGDAKADQAQKVDATGDAK
HMGN2 △12      KAAPKKAAKGKKGSNPAENGDAKADQAQKVDATGDAK
```

B

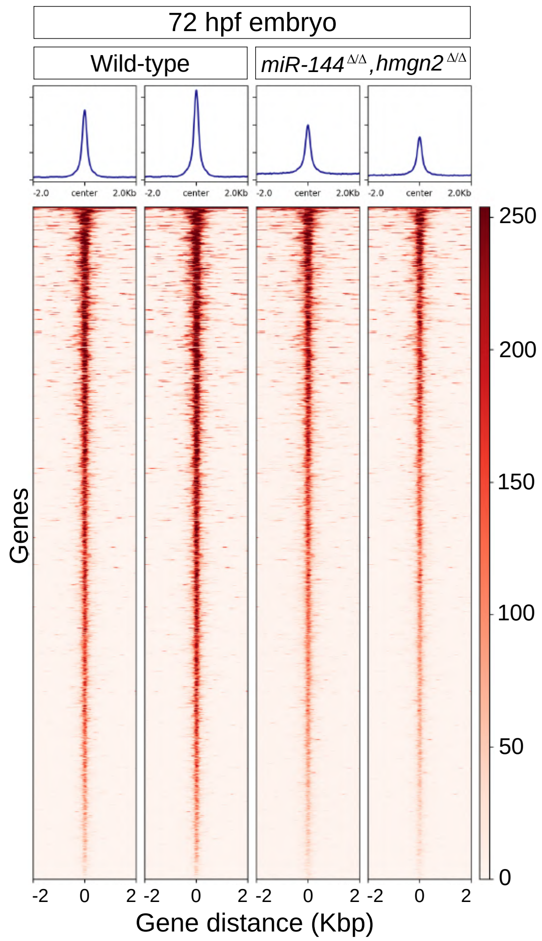

Figure S4. Related to Main Figure 4.

(A) Aminoacid sequence alignment of wild-type and mutant zebrafish Hmgn2. Aligment gap indicated the deletion induced with CRISPR/Cas9.

(B) Heat maps display variations in chromatin accessibility, determined through ATAC-Seq analysis of two separate samples obtained from erythrocytes isolated from peripheral blood at 72 hours post-fertilization (hpf) in both wild-type and double mutant miR-144$^{\Delta/\Delta}$, Hmgn2$^{\Delta/\Delta}$ embryos.

Figure S5

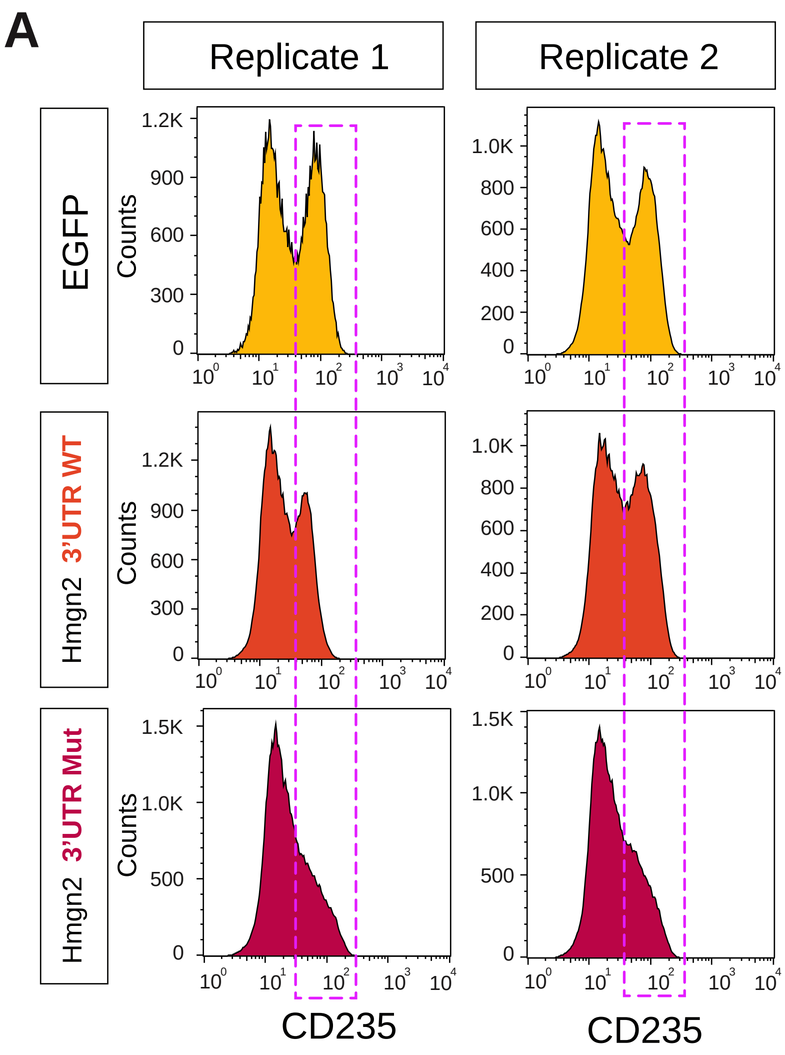

A
Replicate 1
Replicate 2
EGFP
Hmgn2 3'UTR WT
Hmgn2 3'UTR Mut
Counts
CD235

Figure S5. Related to Main Figure 5. Flow cytometry analysis of human erythroid progenitor cells with different levels of HMGN2 activity.

(A) Flow cytometry analysis (FACS) of erythroid cells at 7 days of differentiation to quantify the expression levels of CD235. Pink box indicates the $CD235^+$ population of cells. This data is an independent biological replicate form the data presented in main figure 5G.
