# Supplementary material for: The miR-144/Hmgn2 regulatory axis orchestrates chromatin organization during erythropoiesis
Source: Nat Commun. 2024 May 7;15:3821. doi: 10.1038/s41467-024-47982-2 (PMC11076586; doi:10.1038/s41467-024-47982-2)
Supplement: Supplementary file 3 — Description of Additional Supplementary Files [file 41467_2024_47982_MOESM3_ESM.pdf]

Description of Additional Supplementary Files

File Name: Supplementary Data 1
Description: Table containing the sequence of the oligonucleotides used in this manuscript.
