# Supplementary material for: The miR-144/Hmgn2 regulatory axis orchestrates chromatin organization during erythropoiesis
Source: Nat Commun. 2024 May 7;15:3821. doi: 10.1038/s41467-024-47982-2 (PMC11076586; doi:10.1038/s41467-024-47982-2)
Supplement: Supplementary file 5 — Reporting Summary [file 41467_2024_47982_MOESM5_ESM.pdf]

nature portfolio

Corresponding author(s): NCOMMS-23-34907

Last updated by author(s): Aug 30, 2023

Reporting Summary

Nature Portfolio wishes to improve the reproducibility of the work that we publish. This form provides structure for consistency and transparency in reporting. For further information on Nature Portfolio policies, see our Editorial Policies and the Editorial Policy Checklist.

Statistics

For all statistical analyses, confirm that the following items are present in the figure legend, table legend, main text, or Methods section.

n/a	Confirmed	
☐	☒	The exact sample size (*n*) for each experimental group/condition, given as a discrete number and unit of measurement
☐	☒	A statement on whether measurements were taken from distinct samples or whether the same sample was measured repeatedly
☐	☒	The statistical test(s) used AND whether they are one- or two-sided<br>*Only common tests should be described solely by name; describe more complex techniques in the Methods section.*
☒	☐	A description of all covariates tested
☒	☐	A description of any assumptions or corrections, such as tests of normality and adjustment for multiple comparisons
☐	☒	A full description of the statistical parameters including central tendency (e.g. means) or other basic estimates (e.g. regression coefficient) AND variation (e.g. standard deviation) or associated estimates of uncertainty (e.g. confidence intervals)
☐	☒	For null hypothesis testing, the test statistic (e.g. *F*, *t*, *r*) with confidence intervals, effect sizes, degrees of freedom and *P* value noted<br>*Give P values as exact values whenever suitable.*
☒	☐	For Bayesian analysis, information on the choice of priors and Markov chain Monte Carlo settings
☒	☐	For hierarchical and complex designs, identification of the appropriate level for tests and full reporting of outcomes
☒	☐	Estimates of effect sizes (e.g. Cohen's *d*, Pearson's *r*), indicating how they were calculated

Our web collection on statistics for biologists contains articles on many of the points above.

Software and code

Policy information about availability of computer code

Data collection	N/A
Data analysis	PRISM, R, ImageJ, FloJo, Kallisto (v0.46.1) ,Sleuth (v0.30.0)

For manuscripts utilizing custom algorithms or software that are central to the research but not yet described in published literature, software must be made available to editors and reviewers. We strongly encourage code deposition in a community repository (e.g. GitHub). See the Nature Portfolio guidelines for submitting code & software for further information.

Data

Policy information about availability of data

All manuscripts must include a data availability statement. This statement should provide the following information, where applicable:

- Accession codes, unique identifiers, or web links for publicly available datasets
- A description of any restrictions on data availability
- For clinical datasets or third party data, please ensure that the statement adheres to our policy

All sequencing data were deposited to NCBI as SRA under the following Bioproject identifiers: 1) ATAC-seq data: PRJNA1007738 [https://www.ncbi.nlm.nih.gov/bioproject/PRJNA1007738], 2) RNA-Seq, scRNA-Seq and Quant-Seq data: PRJNA1010662 [https://www.ncbi.nlm.nih.gov/bioproject/PRJNA1010662]. Source data are provided with this paper. Raw images are deposited in Figshare under DOI:10.6084/m9.figshare.25552233. Reasonable requests for resources and reagents should be directed to and will be fulfilled by the Lead Contact, Daniel Cifuentes (dcb@bu.edu).

Research involving human participants, their data, or biological material

Policy information about studies with human participants or human data. See also policy information about sex, gender (identity/presentation), and sexual orientation and race, ethnicity and racism.

Reporting on sex and gender	N/A
Reporting on race, ethnicity, or other socially relevant groupings	N/A
Population characteristics	N/A
Recruitment	N/A
Ethics oversight	N/A

Note that full information on the approval of the study protocol must also be provided in the manuscript.

Field-specific reporting

Please select the one below that is the best fit for your research. If you are not sure, read the appropriate sections before making your selection.

[x] Life sciences [ ] Behavioural & social sciences [ ] Ecological, evolutionary & environmental sciences

For a reference copy of the document with all sections, see nature.com/documents/nr-reporting-summary-flat.pdf

Life sciences study design

All studies must disclose on these points even when the disclosure is negative.

Sample size	Multiple zebrafish embryos from different parents were pooled in each sample to minimize biological variability. Samples sizes were chosen to show reproducible differences between control and mutant/treated samples.
Data exclusions	Representative images of cells, embryos and blots where selected among replicates to be displayed in main figure panels. The data from the other replicates is embedded in quantification plots.
Replication	Experiments were performed at least in triplicates unless otherwise indicated
Randomization	N/A
Blinding	N/A

Reporting for specific materials, systems and methods

We require information from authors about some types of materials, experimental systems and methods used in many studies. Here, indicate whether each material, system or method listed is relevant to your study. If you are not sure if a list item applies to your research, read the appropriate section before selecting a response.

Materials & experimental systems

n/a	Involved in the study	
[ ]	[x]	Antibodies
[ ]	[x]	Eukaryotic cell lines
[x]	[ ]	Palaeontology and archaeology
[ ]	[x]	Animals and other organisms
[x]	[ ]	Clinical data
[x]	[ ]	Dual use research of concern
[x]	[ ]	Plants

Methods

n/a	Involved in the study	
[x]	[ ]	ChIP-seq
[ ]	[x]	Flow cytometry
[x]	[ ]	MRI-based neuroimaging

Antibodies

Antibodies used	anti-RNAP II Phosho-Ser2 (Abcam #5095), anti-rabbit Alexa Fluor 488 secondary antibodies (Jackson Immuno Research Laboratories, #711-545-152), BD Horizon™ BV421 Mouse anti-Human CD235a antibody (#562938) or PE Mouse anti-Human CD235a antibody (#555570), anti-FLAG-M2 magnetic beads (Millipore Sigma #M8823), anti-FLAG-M2 (Millipore Sigma #F7425), anti-HA (Millipore Sigma, #H6908), anti-Actin (Millipore, #MAB1501R), IRDye 800CW Goat anti-mouse (Li-COR, #925-32210), IRDye 680RD Goat anti-rabbit (Li-COR, #926-68071), IRDye 800CW Goat anti-rabbit (Li-COR, #926-32211)
Validation	Manufacturer website providing validation pictures.

Eukaryotic cell lines

Policy information about cell lines and Sex and Gender in Research

Cell line source(s)	Cells were derived from a Boston University library of human sickle cell disease induced pluripotent stem cells (iPSCs)
Authentication	none of the cells were authenticated.
Mycoplasma contamination	Cell lines were tested negatively for mycoplasma
Commonly misidentified lines (See ICLAC register)	N/A

Animals and other research organisms

Policy information about studies involving animals; ARRIVE guidelines recommended for reporting animal research, and Sex and Gender in Research

Laboratory animals	Zebrafish (Danio rerio) embryos were used in this study. 48 and 72-hours old embryos were derived from a mix of wild-type strains incrosses (AB, TU, TL , and NHGRI-1). The sex is not-relevant at this age because sex determination occurs at later stages. When adult zebrafish (>3-month old) were used, the blood was collected from both males and females.
Wild animals	N/A
Reporting on sex	Zebrafish embryos were not discriminated by sex because sex determination takes place at later time points. Collection of peripheral blood from adult fish was sourced from both males and females. Pronephros samples for scRNA-Seq analysis were extracted from adult males. For single-cell sequencing experiment of adult zebrafish pronephros only males (>3-month old) were used.
Field-collected samples	N/A
Ethics oversight	Zebrafish strains were bred, handled, and maintained according to the standard laboratory conditions under IACUC protocol PROTO201800373 at Boston University.

Note that full information on the approval of the study protocol must also be provided in the manuscript.

Flow Cytometry

Plots

Confirm that:

- [x] The axis labels state the marker and fluorochrome used (e.g. CD4-FITC).
- [x] The axis scales are clearly visible. Include numbers along axes only for bottom left plot of group (a 'group' is an analysis of identical markers).
- [ ] All plots are contour plots with outliers or pseudocolor plots.
- [x] A numerical value for number of cells or percentage (with statistics) is provided.

Methodology

Sample preparation	Cells were stained on ice for 25 minutes using the following antibodies: anti-CD235a-PE (BD; #555570) or anti-CD235a-BV421 (#562938)
Instrument	Stratedigm S1000EXI
Software	FlowJo v8.7 (FlowJo, LLC)
Cell population abundance	20000 cells were analyzed for each experiment

Gating strategy

FACS plots shown represent live erythroid cells based on side-scatter/forward-scatter gating.

☐ Tick this box to confirm that a figure exemplifying the gating strategy is provided in the Supplementary Information.
